# Therapeutic effect of valsartan against doxorubicin-induced renal toxicity in rats

**DOI:** 10.22038/ijbms.2019.32871.7851

**Published:** 2019-03

**Authors:** Hai-Xia Liu, Jin Li, Qi-Xiong Li

**Affiliations:** 1 Department of Pharmacology, City College, Wuhan University of Science and Technology, Wuhan, China; 2 Department of Laboratory, Zhongnan Hospital of Wuhan University, Wuhan, China

**Keywords:** Antioxidants, Doxorubicin, Histopathology, Nephrotoxicity, Valsartan

## Abstract

**Objective(s):**

Doxorubicin (DXR)-induces glomerular atrophy and fibrosis in rat kidneys. The objective of the current study was to investigate the protective effects of valsartan on DXR-induced glomerular toxicity and its mechanisms of actions in rats.

**Materials and Methods:**

Male Sprague-Dawley (SD) rats were divided into four groups, and each group contains ten rats. First group was control and was treated with saline only. Treatment groups were injected with DXR (6.5 mg/kg) alone, or intragastric gavage with 10 mg/kg or 20 mg/kg of valsartan after DXR treatment.

**Results:**

Rats treated with DXR only showed significant changes in concentrations of urinary protein, serum creatinine (SCr), and blood urea nitrogen (BUN). Moreover, glomerular structural damages were observed in rats treated with DXR. Valsartan significantly alleviated the effect of DXR. Dramatic elevation in malondialdehyde (MDA), nitric oxide (NO), nitric oxide synthase (NOS) and significant reductions in the levels of reduced glutathione (GSH), glutathione peroxidase (GPx), superoxide dismutase (SOD) were seen after DXR treatment. These effects were effectively ameliorated by co-administration with valsartan.

**Conclusion:**

The findings of our study indicate that valsartan may play an important role in protecting DXR-induced renal toxicity, at least in part, through its antioxidant properties.

## Introduction

Doxorubicin (DXR) has been known to cause damage in rat kidneys, including glomerular capillary permeability increases and glomerular atrophy (1). DXR accumulates in the glomerulus and causes severe glomerular damage, mainly due to oxidative stress (2). Valsartan (VAL) has been reported to be an effective drug to protect against DXR-induced nephrotoxicity. However, the mechanism of protection is not clear, though it has been postulated that antioxidant activities of VAL might be one of the major factors. The present study was carried out to test the effects of VAL on DXR -induced glomerular toxicity through its antioxidant properties in rats. 

Valsartan (VAL, ((S)-N-valeryl-N-[2′-{-(1H-tetrazol-5-yl)

biphenyl-4-yl}-methyl]-valine)) is an oral antihypertensive

agent that inhibits the action of angiotensin II type 1 receptor subtype in a competitive and selective manner, making it an effective antihypertensive medicine (3, 4). Experiments *in vivo* demonstrated that VAL alleviates insulin resistance in chronic renal failure rats (5). Previous studies using rat fibrotic renal tissues have reported that valsartan inhibits the accumulation of dendritic cells (6). In addition, it has been shown that VAL alleviates cyclosporine A-induced tubular toxicity by upregulation of renal glutathione peroxidase (GPx) expression and by altering oxidative stress in rats (7). It has been suggested that valsartan may be a promising therapeutic approach in patients with renal dysfunction (8).

**Figure 1:**
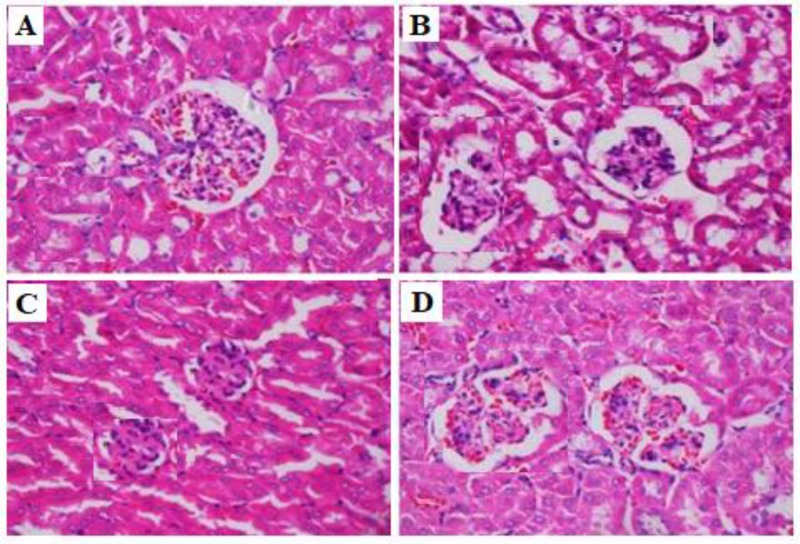
Effects of doxorubicin and valsartan on kidney histology in rats. Light microscopy of kidney tissue (HE stained kidney sections, 400×). (A) Control group: normal glomerular structure. (B) DXR group: glomerular atrophy. (C) DXR+ VAL (10 mg/kg) group: glomerular atrophy alleviation. (D) DXR+ VAL (20 mg/kg) group: glomomerular structure return to normal

**Figure 2:**
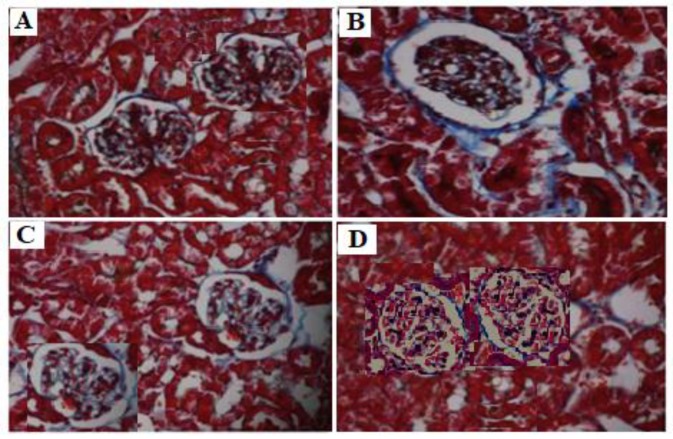
Effects of doxorubicin and valsartan on kidney histology in rats. Light microscopy of kidney tissue (Masson stained kidney sections, 400×). The glomerular fibrotic tissue stained with Maroon is blue. (A) Control group: normal glomerular structure. (B) DXR group: glomerular fibrosis. (C) DXR+ VAL (10 mg/kg) group: glomerular fibrosis alleviation. (D) DXR+ VAL (20 mg/kg) group: glomomerular fibrosis return to normal

**Table 1:** The levels of 24 h urinary protein in rats treated with doxorubicin or doxorubicin plus valsartan

Group	Urinary protein/mg/d		
	10	20	30(d )
Control	0.48±0.12	0.52±0.16	0.56±0.38
DXR	1.08±0.14 **	10.46±0.76 **	18.84±1.46**
DXR + VAL (10mg/kg)	0.88±0.41 #	8.44±1.43 #	12.12±1.24 #
DXR + VAL (20mg/kg)	0.42±0.26##	4.84±0.09##	5.08±0.68##

Data given are the mean ± standard deviations (*n *=10).

VAL: valsartan, DXR: doxorubicin

** Significantly different from control group (*P* <0.01).

# Significantly different from DXR group (*P* <0.05).

## Significantly different from DXR group (*P* <0.01).

**Table 2:** Blood urea nitrogen and serum creatinine concentrations in rats treated with doxorubicin or doxorubicin plus valsartan

Group	BUN/mmol/L	SCr/mol/L
Control	6.8±0.8	64±10
DXR	10.5±0.6**	106±15 **
DXR + VAL (10mg/kg)	8.4±0.4 #	87±14 #
DXR + VAL (20mg/kg)	6.4±0.4##	68±10 ##

Data given are the mean ± standard deviations (*n *=10).

VAL: valsartan, DXR: doxorubicin, BUN: blood urea nitrogen, SCr: serum creatinine

** Significantly different from control group (*P* <0.01).

# Significantly different from DXR group (*P* <0.05).

## Significantly different from DXR group (*P* <0.01).

**Table 3:** Kidney reduced glutathione, malondialdehyde, nitric oxide levels and glutathione peroxidase, superoxide dismutase, nitric oxide synthase activities in rats treated with doxorubicin or doxorubicin plus valsartan

Group	GSH	GPx	MDA	SOD	NO	NOS
	mol/g	nmol/min^/^mg/^/^prot	nmol/mg/prot	nmol/min/g	μmol/g/prot	nmol/min^/^g/prot
Control	1.18±0.02	134.4±2.2	0.68±0.06	24.4±1.3	0.148±0.012	0.362±0.012
DXR	0.26±0.04**	22.7±3.1**	8.26±0.08 **	10.4±1.4**	0.640±0.016**	0.812±0.013 **
DXR + VAL (10mg/kg)	0.64±0.02 #	46.1±1.7#	1.44±0.17#	18.8±2.4#	0.426±0.020#	0.630±0.021#
DXR + VAL (20mg/kg)	1.16±0.03##	133.4±0.8##	1.08±0.04 ##	22.8±2.2 ##	0.1632±0.023##	0.364±0.014 ##

Data given are the mean ± standard deviations (*n *=10).

VAL: valsartan, DXR: doxorubicin, GSH: reduced glutathione, MDA: malondialdehyde, SOD: superoxide dismutase, GPx: glutathione peroxidase, MDA: malondialdehyde, NO: nitric oxide, NOS: nitric oxide synthase

** Significantly different from control group (*P* <0.01).

# Significantly different from DXR group (*P* <0.05).

## Significantly different from DXR group (*P* <0.01).

## Materials and Methods

All animal studies were approved by the Wuhan University Animal Care Committee. In this study, a total of 40 healthy male Sprague. Dawley rats (8-week-old weighing 180±20 g) were used. The animals were supplied by Wuhan University Experimental Research Centre, Wuhan, China. The animals were maintained in standard cages at room temperature (20-24 ^°^C) and regular light cycle (12 light/12 dark) with free access to food and water. Animals were divided into four groups, with each group containing 10 rats: I), control group: animals received an intravenously (IV) injection of 0.9% saline via a tail vein, followed by distilled water (10 ml/kg) daily through an intragastric gavage for 30 days; II) DXR only group: rats received IV injection of a single dose of DXR (6.5 mg/kg) via a tail vein, followed by distilled water (10 ml/kg) through an intragastric gavage daily for 30 days; III). DXR + VAL groups: rats received a single dose of DXR (6.5 mg/kg) injection through a tail vein, followed by daily administration of VAL (10 mg/kg, or 20 mg/kg for 30 days intragastrically (3). At the 10^th^, 20^th^ and 30^th ^day after VAL administration, urines were collected for 24 hr to measure the urinary protein content. Four hr after the last drug administration, all animals were sacrificed after sodium pentobarbital (45 mg/kg) anesthetization. Blood samples were collected to measure serum creatinine (SCr) and blood urea nitrogen (BUN) concentrations. Kidneys from both sides were rapidly removed and sectioned at 4 µm thickness for histological examination. The remaining tissues after histological slices were homogenized in Tris–HCl buffer (0.05 mol/l Tris–HCl, 1.15% KCl, pH 7.4), using a Polytron homogeniser. The homogenate was centrifuged at 18,000×*g *(+4 ^°^C) for 30 min and supernatant was stored at -80 ^°^C until further used for biochemical analysis.

Urinary protein content was analyzed using the sulfosalicylic acid colorimetric method as described previously (9). BUN and SCr levels were assayed using diacetyl monoxime and basic picric acid as substrates, respectively. Malondialdehyde (MDA) concentration was determined using a method based on the reaction with thiobarbituric acid (10). Superoxide dismutase (SOD) activity was determined using pyrogallol as a substrate (11). Reduced glutathione (GSH) content was assayed colorimetrically using the DTNB Ellman reagent (12). Glutathione peroxidase (GPx) was analyzed by an enzymatic method described previously (13). Nitric oxide (NO) concentration and nitric oxide synthase (NOS) activity were determined by a colorimetric method based on Griess reaction (14). Total protein was measure by Lowry method using bovine serum albumin as a standard (15). 

At the end of the experiment the kidneys from both sides were rapidly removed, immersed in 4% formaldehyde for fixation and embedded in paraffin for sectioning. A microtome was used to cut sections at 4 μm thickness, and the sections were mounted on a glass slide and stained with haematoxylin/eosin (HE) or Masson, respectively. The glomerular pathological changes were viewed under light microscopy. The glomerular fibrotic tissue stained with Masson is blue. 

All values were obtained from 10 animals in each groups and expressed as mean ± standard deviations. The statistic differences were done by Student’s *t*-test and one-way analysis of variance (ANOVA) using SPSS software 17.0 (Released Aug. 23, 2008), Chicago, USA.A *P-*value of < 0.05 was considered statistically different. 

## Results

As demonstrated in Table 1, urinary protein 10, 20 and 30 days after DXR treatment was significantly increased (*P*<0.01). These changes in urinary proteins were significantly reduced in a dose dependent manner by co-administration of VAL (10 and 20 mg/kg) (*P*<0.05 and *P*<0.01, respectively). Significant increases in BUN and SCr concentrations were observed in rats treated with DXR alone (*P*<0.01, compared with control). However, these changes were effectively reversed by co-administration of VAL at both 10 and 20 mg/kg doses (Table 2). Figure 1a, and Figure 2a showed that control animals had normal glomerular structures in renal tissues. However, significant disorganization of glomerular structure, glomerular atrophy and fibrosis were observed in rats treated with DXR (Figure 1b, Figure 2b). Glomerular atrophy and fibrosis were ameliorated in VAL (10 mg/kg) treated group compared to the DXR treated group (Figure1c, Figure 2c). VAL (20 mg/kg) significantly reduced glomerular atrophy and fibrosis induced by DXR, with glomomerular structure returned to normal (Figure 1d, Figure 2d).

Results of the antioxidative properties of VAL were summarized in Table 3. DXR treatment resulted in lowered levels of GSH, GPx, and SOD in kidney tissues, while MDA, NO levels and the activity of NOS were higher (*P*<0.01). DXR-induced changes in levels and activities of MDA, NO, NOS, GSH, SOD and GPx were all dramatically alleviated by co-administration of Val at 10 and 20 mg/kg (*P*<0.01).

**Discussion**

Among the clinically used angiotensin II type 1 receptor subtype blockers, VAL is the most frequently used antihypertensive drug. Apart from its role as an antihypertensive drug, VAL also has beneficial effect to the kidney and is also used in supplemental therapies for kidney diseases (8, 16). Several previous reports indicate that VAL plays a pivotal role in protecting against progressing renal tubule injury (7, 17, 18). However, the effect of VAL on glomerular injury remains unknown. 

DXR is an effective agent in treating a variety of solid tumors but like any other anti-tumor drugs, DXR has significant adverse effects in kidneys (1). DXR accumulates in the glomerulus and causes severe kidney damage. The mechanisms by which DXR causes glomerular toxicity have not been fully elucidated. However, some previous reports have demonstrated that t reactive oxygen species and free radicals are major contributing factors for the nephrotoxicity induced by DXR (2, 19, 20). This study has confirmed that VAL treatment after the administration of DXR significantly changed urinary protein, BUN, SCr, and notably ameliorated renal histopathological injury. Therefore, it is suggested that VAL has protective effects on DXR-induced glomerular toxicity. 

Several studies have shown that a NO, at physiological concentrations, is important for maintaining normal renal function (21). However, excessive production of NO becomes detrimental and plays an important role in the pathophysiology of acute renal failure by inducing oxidative stress and tissue damage (22). Our data showed that DXR markedly increased NO and NOS levels in kidney tissues and VAL significantly alleviated these increases. Because of role that NO and NOS play in acute renal failure in the rat, it is plausible to postulate that inhibition of NO and NOS during DXR treatment may offer protection against DXR nephrotoxicity.

MDA is often measured as an indicator of lipid peroxidation and its increase is directly linked to free radical damage to glomerular basement membrane. SOD enzyme catalyzes the dismutation of O2^•-^ to H_2_O_2_ and molecular oxygen (O_2_), while GPx catalyzes the degradation of H_2_O_2_ to O_2_ and H_2_O (23). In the present study, we found that DXR treatment caused a dramatic elevation in MDA content and significant reduction in GSH content, GPx, and SOD activities.. VAL significantly alleviated changes in these parameters induced by DXR. The therapeutic effect of VAL observed in this study may be due to its antioxidant and free radical scavenging abilities because it effectively reversed the reduction of GSH and the decrease in GPx and SOD antioxidant activities induced by DXR. A recent study showed that VAL may attenuate the nephrotoxic side effect of cyclosporine via upregulation renal GPx expression and altering oxidative stress (7). Our study confirmed that VAL alleviates DXR-induced glomerular toxicity due to its ability to increase antioxidant activities.

## Conclusion

The results of our present study indicate that DXR causes glomerular toxicity by oxidative stress. VAL may play a renoprotective role in DXR-induced glomerular toxicity, at least in part, through its antioxidant properties. 

## Conflicts of Interest

The authors state no conflict of interest.
